# Acute Renal Failure Revealing Primary Renal Lymphoma With Bone Involvement: A Rare Case

**DOI:** 10.7759/cureus.105900

**Published:** 2026-03-26

**Authors:** Ilyass Laaribi, Safaa Kachmar, Samia Berrichi, Ikram Zaid, Houssam Bkiyar

**Affiliations:** 1 Intensive Care Unit, Mohammed VI International University Hospital, Faculty of Medicine and Pharmacy of Oujda, Mohammed First University, Oujda, MAR

**Keywords:** acute kidney injury, bone involvement, case report, diffuse large b-cell lymphoma, primary renal lymphoma

## Abstract

Primary renal diffuse large B-cell lymphoma is rare, and its presentation with acute kidney injury (AKI) due to bilateral renal infiltration, occasionally associated with bone involvement, is exceptionally uncommon. We report the case of a 46-year-old man with a history of low back pain, previously misdiagnosed as lumbosacral radiculopathy, who was admitted for severe AKI. Imaging revealed bilaterally enlarged kidneys with multiple hypodense parenchymal lesions, without evidence of extrarenal disease. Renal biopsy confirmed diffuse large B-cell lymphoma, and further evaluation revealed secondary bone involvement. The patient was promptly referred for chemotherapy to preserve renal function. This case highlights the diagnostic challenges of primary renal lymphoma (PRL), as its clinical and radiological presentation is nonspecific; the disease should be suspected in atypical AKI, emphasizing that early histological diagnosis is crucial to ensure timely and effective management.

## Introduction

Renal involvement in lymphomas is relatively common in advanced systemic forms; however, it generally represents a secondary localization or a complication of the disease. Primary renal lymphoma (PRL), on the other hand, is extremely rare and is defined as exclusive renal involvement without other sites of disease at the time of diagnosis [1]. In the literature, PRL presenting with acute kidney injury (AKI) is considered exceptional, as most reported renal involvements are secondary to systemic lymphomatous disease [2]. It is most frequently observed in the setting of non-Hodgkin lymphomas, particularly diffuse large B-cell lymphoma. The rarity of PRL is partly explained by the near absence of organized lymphoid tissue within the renal parenchyma. Consequently, most renal involvements are secondary, resulting either from hematogenous dissemination or from direct extension from retroperitoneal lymph nodes [3].

Clinical and radiological manifestations are generally nonspecific, and confirmation most often relies on histological examination [4]. Early recognition of this entity is essential, as appropriate chemotherapy may lead to significant improvement, or even recovery, of renal function [5].

Herein, we report a case of renal lymphoma revealed by AKI.

## Case presentation

We present the case of a 46-year-old man with a history of low back pain, initially misdiagnosed as lumbosacral radiculopathy and treated with nonsteroidal anti-inflammatory drugs (NSAIDs) for several months, who presented to the emergency department with one week of abdominal pain, nausea, vomiting, and asthenia.

On examination, he was alert, hemodynamically stable, and apyretic, with right flank tenderness on abdominal palpation.

Laboratory evaluation revealed severe AKI (Table 1). Immediate potassium-lowering measures were initiated, and urgent hemodialysis was performed.

**Table 1:** Blood and urine analysis on admission.

Test	Result	Reference range
White blood cell	7.6	4-10 × 10³/µL
Hemoglobin	9.1	14-17 g/dl
Platelet count	257	150-500 × 10³/µL
Urea	32.2	2.5-7.5 mmol/L
Creatinine	1,106	53-115 µmol/L
Glomerular filtration rate (GFR)	4.5	> 90 mL/min/1.73 m²
Total protein	70	60-80 g/L
Sodium	137	135-145 mmol/L
Potassium	7	3.5-5.1 mmol/L
Chloride	101	95-110 mmol/L
Calcium	93	86-105 mg/L
Lactase dehydrogenase	308	135-225 UI/L
C-reactive protein (CRP)	56.1	0-5 mg/L
Urine leukocytes	6	< 10 × 10³/µL
Urine erythrocytes	12	< 5 × 10³/µL

**Figure 1:**
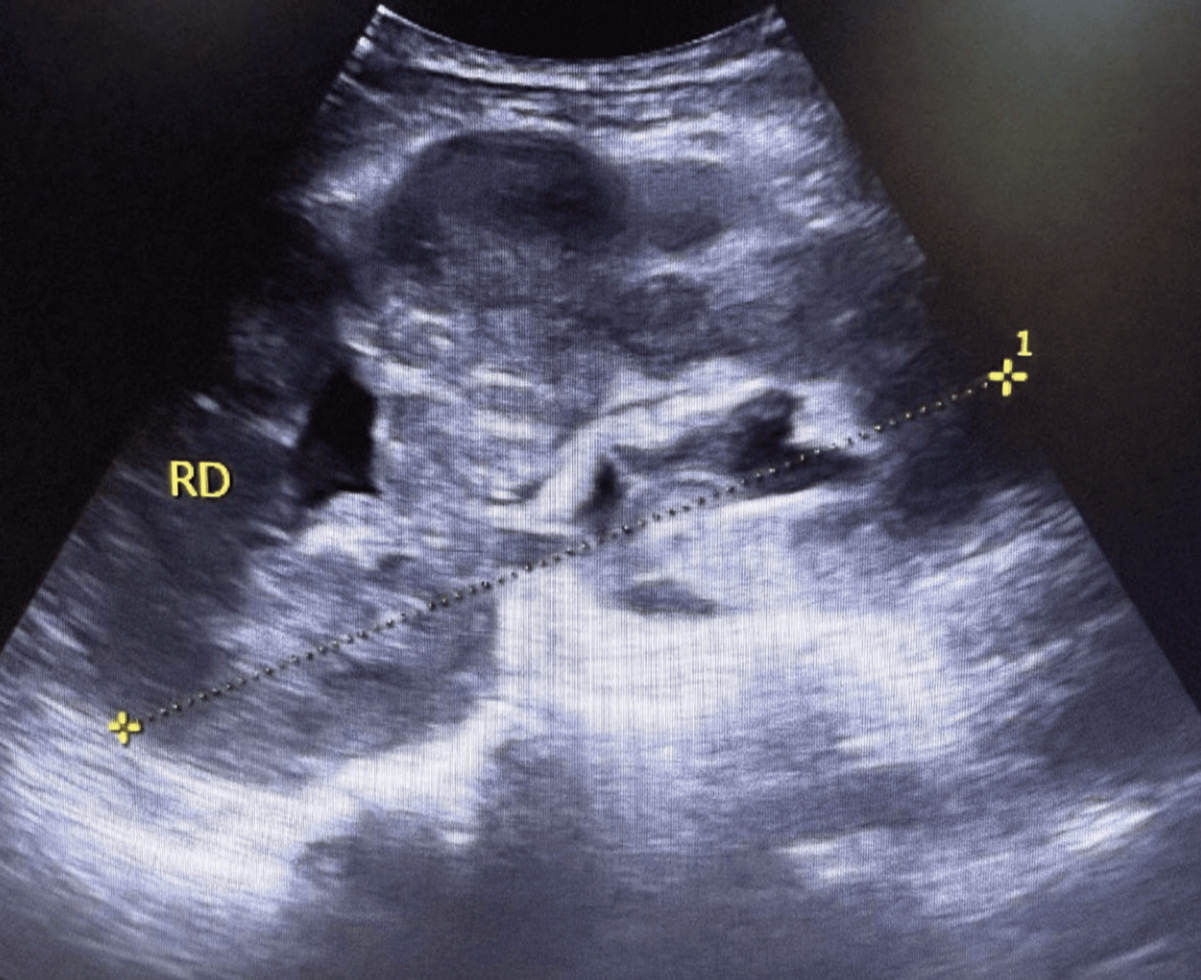
Ultrasound of the right kidney demonstrating renal enlargement with poor corticomedullary differentiation, without evidence of calculi.

**Figure 2:**
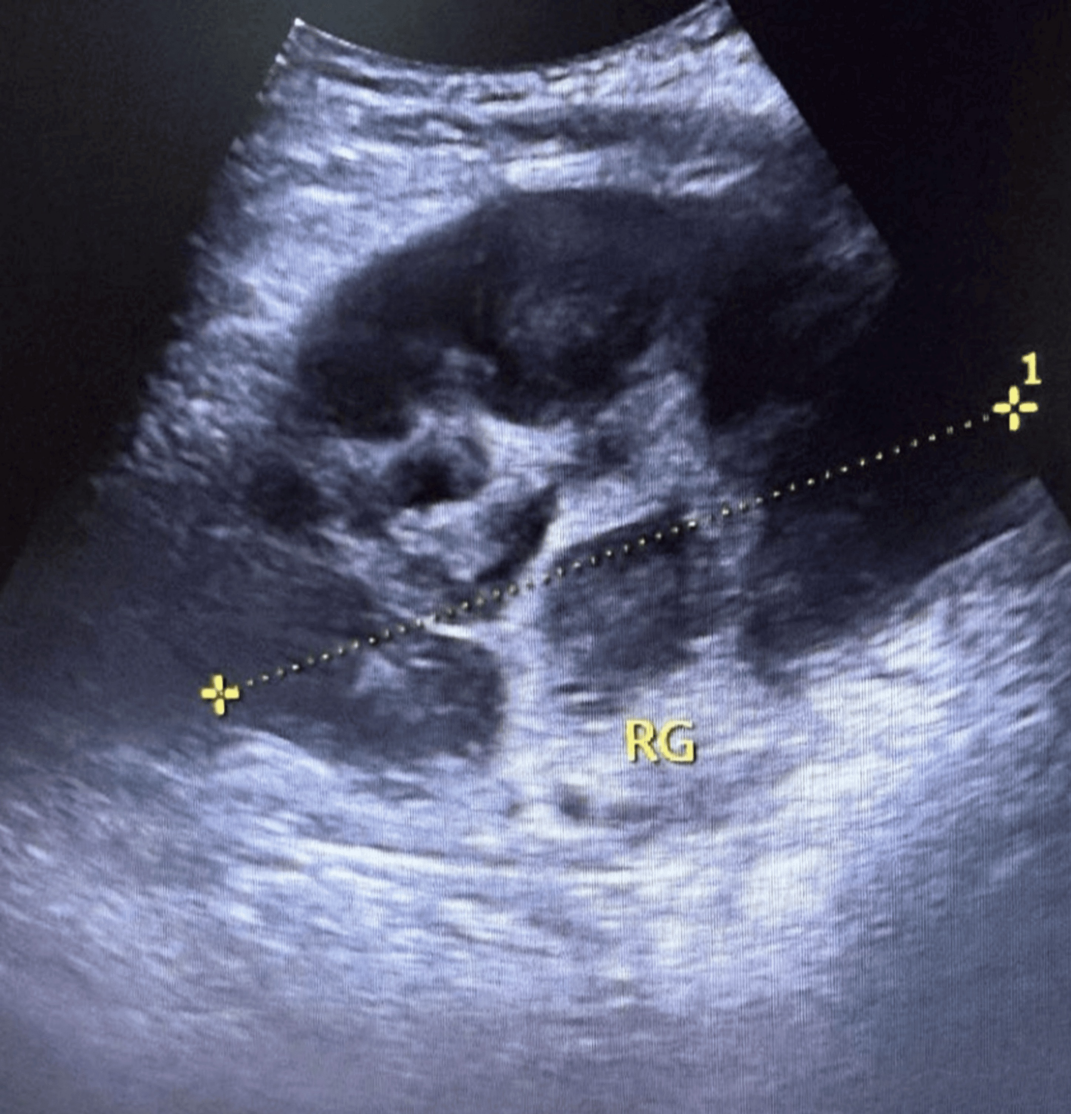
Ultrasound of the left kidney demonstrating renal enlargement with poor corticomedullary differentiation, without evidence of calculi.

**Figure 3:**
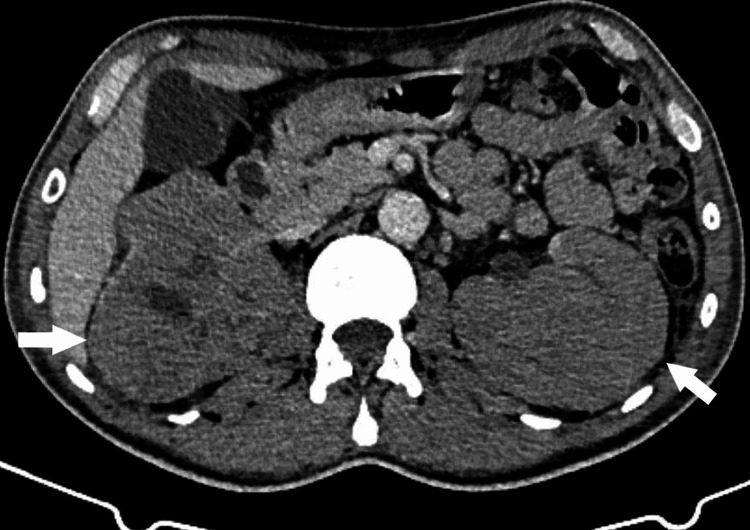
Axial abdominal CT scan showing bilateral nephromegaly with hypodense parenchymal infiltrates, without extracapsular extension, compatible with renal lymphoma.

**Figure 4:**
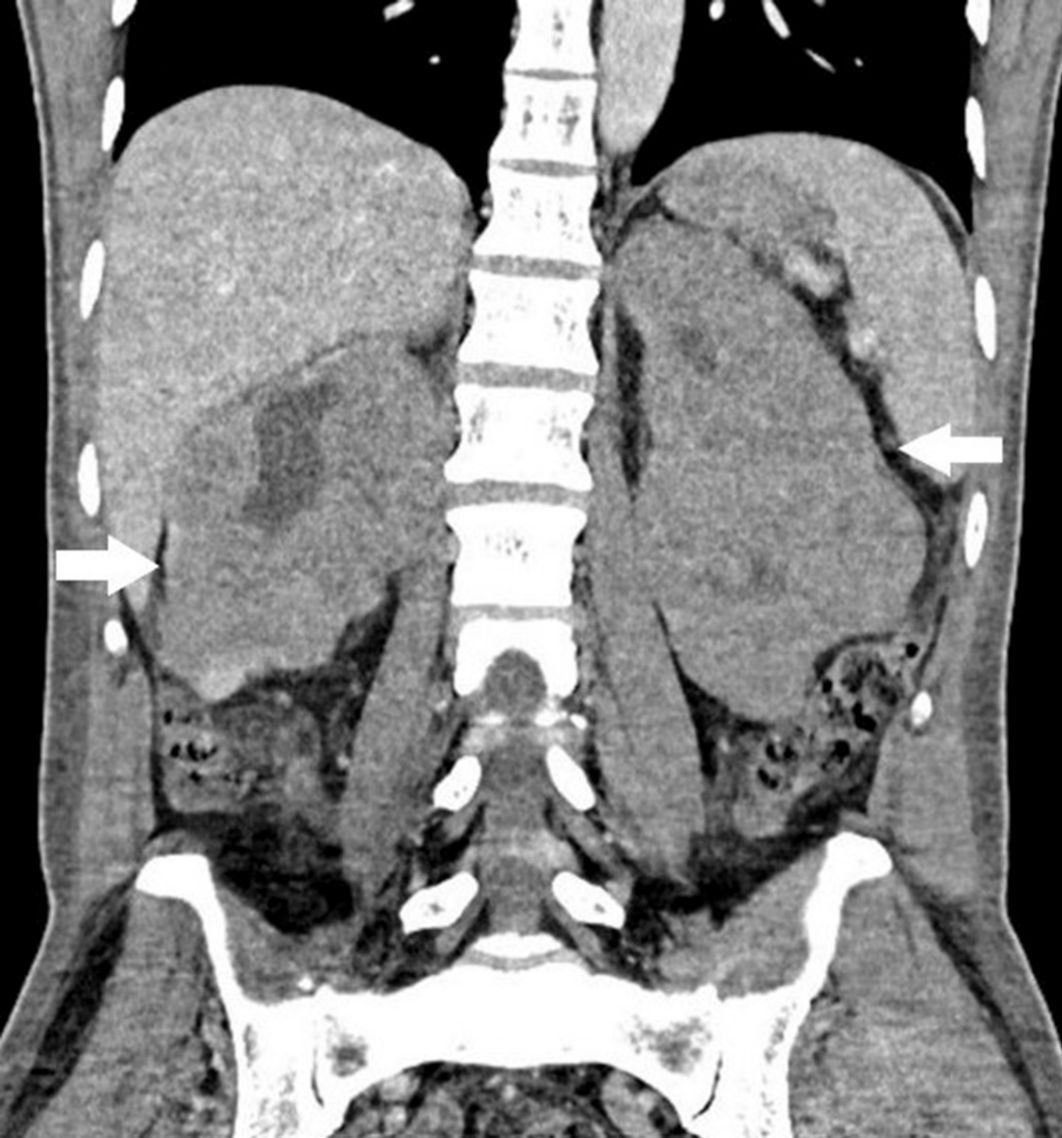
Coronal abdominal CT scan showing bilateral nephromegaly with hypodense parenchymal infiltrates, without extracapsular extension, compatible with renal lymphoma.

Renal ultrasound, performed to rule out obstruction, showed bilaterally enlarged kidneys with poorly differentiated parenchyma, without stones or hydronephrosis (Figures 1-2). Subsequent contrast-enhanced CT revealed multiple hypodense, mildly enhancing parenchymal lesions in both kidneys (right: 12 cm, left: 16 cm), without extracapsular extension. No retroperitoneal or lombo-aortic lymphadenopathy, hepatosplenomegaly, or other organ involvement was observed (Figures 3-4).

**Figure 5:**
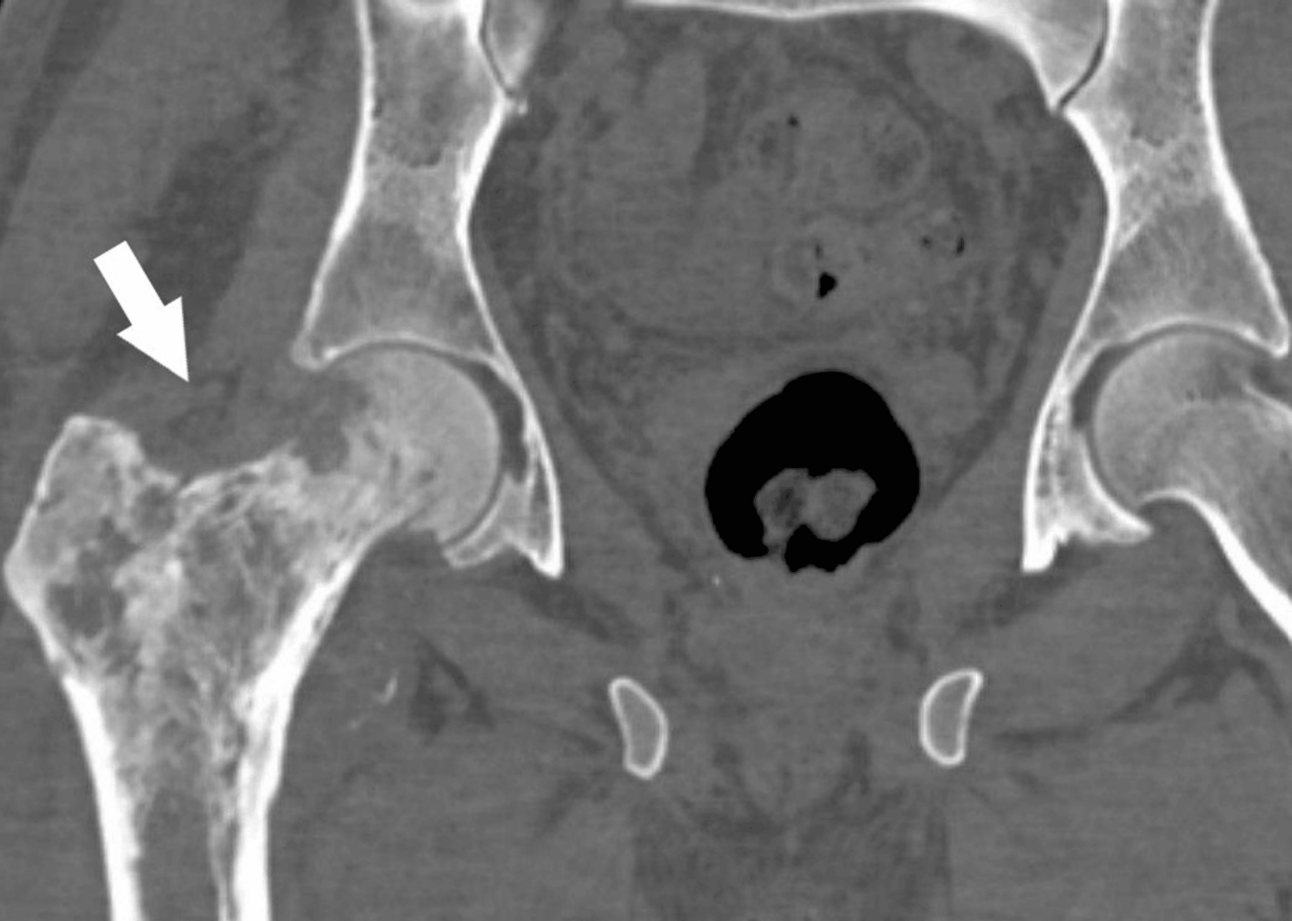
Coronal pelvic CT scan showing secondary lesions of the right femoral neck.

**Figure 6:**
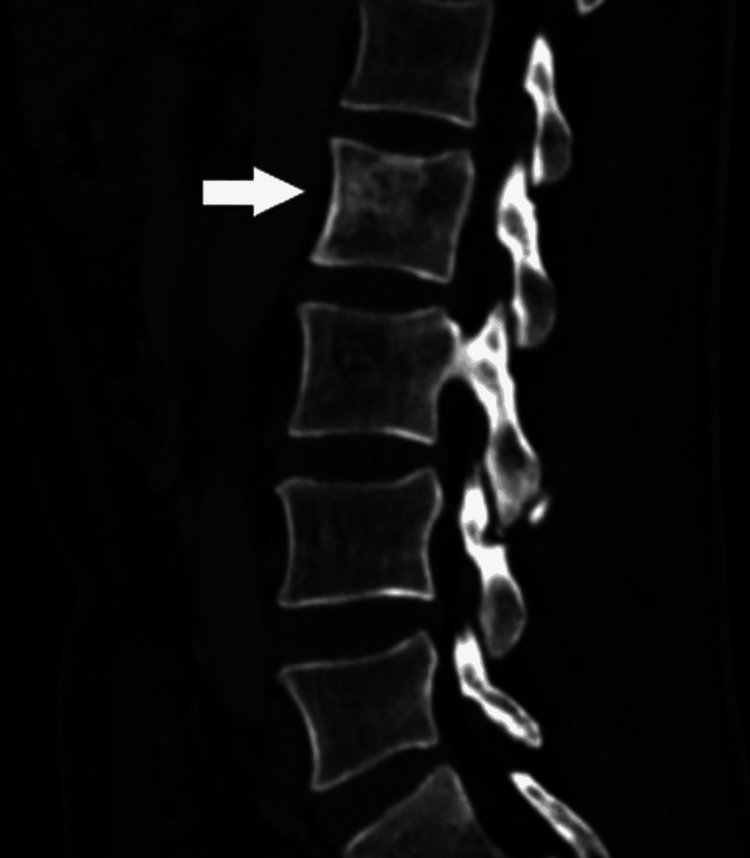
Sagittal abdominal CT scan showing lytic lesions of the vertebral body of the second lumbar vertebra.

Renal biopsy with immunohistochemical confirmed a diffuse large B-cell lymphoma with CD20 (+), CD3 (-), CD5 (-), CD10 (-), CD23 (+), BCL6 (+), BCL2 (-), and Ki-67 (90%). Staging identified secondary bone lesions involving the right femoral neck (Figure 5) and the L2 vertebral body (Figure 6). HIV serology was negative. According to the Ann Arbor classification, the disease was stage IV E, consistent with primary kidney bilateral diffuse large B-cell lymphoma, renal lymphoma, and bone metastasis. The patient was promptly referred to a specialized center for initiation of chemotherapy and had already started treatment at the time of manuscript preparation.

## Discussion

PRL is a rare entity that has long remained controversial due to the absence of lymphatic tissue within the renal parenchyma. It is defined as a non-Hodgkin lymphoma that initially develops in the kidney in the absence of extrarenal lymphomatous involvement at the time of diagnosis [6]. Historically, the diagnosis has relied on the criteria proposed by Stallone et al., which include predominant renal involvement at the time of clinical presentation, the absence of significant extrarenal disease, histological confirmation of lymphomatous infiltration on renal biopsy, and improvement in renal function following the initiation of lymphoma-specific therapy [7].

PRL is an extremely rare clinical entity, accounting for less than 1% of extranodal lymphomas [8]. Diffuse large B-cell lymphoma is the most frequently reported histopathological subtype [9], which was also the case in our patient.

AKI in lymphomas may result from direct infiltration of the renal parenchyma by lymphomatous cells, leading to disruption of renal architecture and impairment of glomerular filtration. This mechanism is most commonly observed in aggressive lymphomas or those with a high tumor burden, highlighting the importance of early histological diagnosis to guide appropriate management [10].

The clinical presentation of PRL is often nonspecific, including flank or abdominal pain, hematuria, fatigue, fever, or weight loss. AKI may also be observed in some cases. Due to these nonspecific symptoms, this condition may mimic other renal diseases, which can delay the diagnosis [11].

Imaging, particularly computed tomography (CT), is the initial investigation used to detect renal lesions suggestive of lymphoma. However, as radiological findings are not specific, the definitive diagnosis relies on histopathological and immunohistochemical examination obtained through renal biopsy or surgical sampling [12].

Literature data also indicate that renal involvement may be associated with unusual sites, with bone involvement in renal lymphoma being among the rarest, with only a few dozen cases reported [13]. This adds additional significance to our case. In such situations, associated bone involvement may present with bone or spinal pain that can mimic lumbosciatica, potentially delaying the diagnosis. In our patient, right flank pain radiating to the lower limb had been present for several months and was initially misattributed to lumbosciatica, treated with NSAIDs. Prolonged use of these medications, known for their nephrotoxic potential, may have contributed to the worsening of renal dysfunction.

Management is based on systemic chemotherapy. The R‑CHOP (rituximab, cyclophosphamide, doxorubicin, vincristine, and prednisone) regimen has historically been the standard first‑line treatment for diffuse large B‑cell lymphoma, achieving durable remissions in a majority of patients [14].

In our case, the diagnosis was established promptly, and treatment was initiated early in the hope of achieving recovery of renal function.

## Conclusions

This case highlights the importance of considering PRL in the differential diagnosis of unexplained AKI with bilateral nephromegaly. A thorough diagnostic approach, including imaging and histological confirmation, is essential to avoid therapeutic delays in these rare but potentially serious presentations.
